# Health Tract No. 336

**Published:** 1868-10

**Authors:** 


HALL'S JOURNAL OF HEALTH. OCTOBER. 1868.

HEALTH TRACT No. 336.

### HOBBY HORSICAL PARENTS.

knew a mother whose fanaticism in the virtues of cold water were such, that she gave her first born a drenching every morning, until it was fairly thrown into convulsions at the very sight of the preparations ; finally dying of epilepsy, after years of suffering.

Frederika Bremer says that her father nearly starved his children to death, under the influence of vagaries in reference to keeping down the animal, and elevating the spiritual nature, by means of a spare diet.

Under the influence of the hallucination that the very first indication of stubbornness on the part of the child should be subdued by all means, a man of education, in the year of grace 1866, beat his son, of two years old, to death with a shingle, because the child would not say its prayers.

A parent discovered that a little child of five years of age was afraid to sleep in a room alone ; and thinking it a mere notion, put the little innocent to bed, put out the light, locked the door and went away ; on visiting the room late at night, the child was found to have died in a fit ; the eyes had started from the sockets, as it were, as if the poor little thing had been horror struck.

Another barbarism is compelling children to eat fat meat, or lean meat or any other article of food, for which there is not only no relish, but an unconquerable antipathy. The instincts of a child should be respected, because they are implanted in its very nature for its well being, as in the animal creation ; might as wisely try to make a kitten eat white beans or compel a chicken to drink salt water ; never war against the instincts of the child ; lead rather than drive ; persuade, rather than punish ; convince, rather than convict ; loose your right arm rather than take advantage of its unresisting helplessness ; bear rather than beat, remembering that "of such is the kingdom of Heaven."

